# Mitochondrial Oxidative Stress and Energy Metabolism: Impact on Aging and Longevity

**DOI:** 10.1155/2021/9789086

**Published:** 2021-08-04

**Authors:** Ravirajsinh N. Jadeja, Pamela M. Martin, Wei Chen

**Affiliations:** ^1^Department of Biochemistry and Molecular Biology, Augusta University, Georgia, USA; ^2^Department of Ophthalmology, Medical College of Georgia, Augusta University, Augusta, GA 30912, USA; ^3^James and Jean Culver Vision Discovery Institute and Medical College of Georgia at Augusta University, Augusta, GA, USA; ^4^Department of Food Science and Nutrition, National Engineering Laboratory of Intelligent Food Technology and Equipment, Zhejiang Key Laboratory for Agro-Food Processing, Zhejiang University, Hangzhou 310058, China

Understanding better processes governing normal aging and the pathogenesis of age-related conditions is essential to potential lifespan extension and/or improvement of quality of life in the geriatric population. Mitochondria are key players in the process of aging because of their critical role in the regulation of bioenergetics, oxidative stress, and cell death [1, 2]. Thus, therapeutic strategies targeted at minimizing oxidative stress and maintaining healthy mitochondrial energy metabolism for productive aging are of noticeable interest to aging researchers. The maintenance of an adequate supply of energy during aging is essential for cellular repair, homeostatic mechanisms, and mitochondrial biogenesis [3, 4]. Numerous studies have shown mitochondrial bioenergetic deterioration to be an important factor in normal physiological aging and in the pathogenesis of age-related ailments in various cell and tissue types [5, 6], with reactive oxygen species- (ROS-) induced mitochondrial DNA damage being a prominent factor [7]. Furthermore, epigenetic modifications have been shown to greatly influence oxidative stress and mitochondrial dysfunction during aging [8]. Related to the above, evidence from recent studies highlights the importance of alterations in the metabolism of nicotinamide adenine dinucleotide (NAD), a coenzyme central to cellular metabolism and epigenetic regulation, in aging [9, 10]. This Special Issue aims to provide recent updates on the role of mitochondrial oxidative stress and energy metabolism in aging and longevity. Following the stringent peer review of many submitted manuscripts, 17 articles were incorporated for publication in this special issue. The article collection for this Special Issue can be subdivided into the following major categories: cardiovascular, retinal and neurological aging, mitochondrial ROS and energy metabolism, and hyperglycemia, as detailed more closely below.

This issue contains two research manuscripts and two review articles focused on cardiovascular aging. The study by E. Qaed et al. evaluated the role of phosphocreatine (PCr) on mitochondrial respiratory function in diabetic cardiomyopathy (DCM). The pretreatment with PCr was found to be effective against cardiac damage in experimental diabetic mice. Another article by Y. Qiao et al. focused on the protective role of capsaicin (CAP) in regulating mitochondrial function in anoxic or anoxic/reoxygenated cardiomyocyte injury. CAP-mediated upregulation of 14-3-3*η*, a protective phosphoserine-binding protein in cardiomyocytes, ameliorated mitochondrial function caused by a disruptive redox status and an impaired ETC (electron transport chain). The other two review articles focused on the role of mitochondrial ROS and mitophagy in maintaining mitochondrial dynamics during cardiovascular aging and collectively provide an excellent review of the existing literature on the field of cardiac aging. Further and importantly, a thorough discussion of potential future therapeutic avenues is also provided.

This special issue also contains three research articles focusing on the role of the mitochondrial function in cerebral injury and aging. The study by Y. Li et al. evaluated the proteomic profile of the aging mouse brain to identify proteins involved in mitochondrial dysfunction and oxidative damage. The proteomic changes of young (4-month) and aged (16-month) B6129SF2/J male mouse hippocampus and cerebral cortex were investigated. Compared with the young animals, 390 hippocampal proteins (121 increased and 269 decreased) and 258 cortical proteins (149 increased and 109 decreased) changed significantly in the aged mice. Bioinformatic analysis indicated that these proteins are mainly involved in mitochondrial function, oxidative stress, synapses, ribosomes, cytoskeletal integrity, transcriptional regulation, and GTPase function. Reutzel et al. performed a longitudinal evaluation of the cerebral mitochondrial function and cognitive performance in aging female NMRI mice. These authors measured brain mitochondrial function, cognitive performance, and molecular markers every 6 months until mice reached the age of 24 months. During the physiological aging process, several changes in cognitive performance, mitochondrial brain energy metabolism, and mRNA expression of genes involved in mitochondrial biogenesis were detected in a longitudinal study over a period of 24 months. Most of the impairments on cognition and mitochondria bioenergetics were detected starting at the age of 18 months, which shows that aged NMRI mice are an appropriate model to study the neurological aging process. Another study by W. Chen et al. evaluated the efficacy of mitoquinone (MitoQ), a newly developed selective mitochondrial reactive oxygen species (ROS) scavenger against intracerebral hemorrhage (ICH) in mice. They demonstrated that the selective mitochondrial ROS scavenger MitoQ can attenuate white matter injury and improve neurological impairment after ICH and supported the potential future use of MitoQ as a therapeutic agent for neuroprotection after ICH.

Review articles from our research group and P. G. Sreekumar et al. provide recent updates on retinal aging. We highlighted specifically the importance of NAD^+^, a coenzyme that participates in various energy metabolism pathways, including glycolysis, *β*-oxidation, and oxidative phosphorylation, in addition to being a required cofactor for important enzymes such as (ADP-ribose) polymerases (PARPs) and sirtuins, with emphasis on the relevance of the above to metabolism in the aging retina and in retinal degeneration. We also discussed possible therapeutic avenues to improve energy metabolism in the aging retina. Alternately, P. G. Sreekumar et al. focused on the topic of ocular senescence. This review provided an overview of the types of senescence, pathways of senescence, and senescence-associated secretory phenotype (SASP). Furthermore, the authors also discussed the role of mitochondria in ocular senescence and possible therapeutic avenues.

This issue also contains several articles focused on understanding the associations between oxidative stress, energy metabolism, and mitochondrial function under various physiological conditions. Y. Du et al. showed that knockdown of mitochondrial antiviral signaling proteins (MAVS) alleviated radiation-induced mitochondrial dysfunction, downregulated the expression of proapoptotic proteins, and reduced the generation of ROS in cells after irradiation. L. Zhao and colleagues showed that autophagy deficiency impairs the antioxidant defense system and could play an important role in the process of aging. In another article by A. Keller et al., it was reported that (–)-epicatechin modulates mitochondrial redox in vascular cell models of oxidative stress. The other two research articles and one review article provided important information related to energy metabolism in various organ systems. C. X. Li et al. reported that inhibition of aldose reductase accelerates liver regeneration by regulating energy metabolism and could serve as a therapeutic target. Using *Caenorhabditis elegans* as an experimental system, B. Dilberger et al. reported that mitochondrial oxidative stress impairs energy metabolism and reduces stress resistance and longevity. The authors further proposed that paraquat-treated *C. elegans* could be a readily accessible in vivo model for mitochondrial dysfunction as it displays the characteristics of oxidative stress, restricted energy metabolism, and reduced stress resistance and longevity. Lastly, the review article by S. Zhang et al. summarized the importance of adropin in the crosstalk between energy regulation and immune regulation.

Finally, this special issue also contains one basic and one clinical research study related to diabetes. L. Lyu et al. evaluated the relationship between leukocyte telomere length (LTL) and mitochondrial DNA copy number (mtDNAcn) in a noninterventional rural community of China with different glucose tolerance statuses. Based on their observation, the authors concluded that tumor necrosis factor-*α* (TNF*α*) may be considered a potential therapeutic target against aging-related disease in hyperglycemia, whereas H. Su et al. demonstrated that andrographolide exerted a glucose-lowering effect through strengthening the intestinal barrier function and increasing the microbial species of *A. muciniphila*. The authors of the latter study also suggested that it might be plausible to prevent type-2 diabetes by regulating gut barrier integrity and shaping intestinal microbiota composition.

In summary, we hope that readers find this special issue to be as interesting as it is important. The overarching goal of the review articles and original research manuscripts assembled herein is to advance the current knowledge of the mechanisms governing normal aging and the development and progression of age-related diseases by providing new insights into the role of mitochondrial oxidative stress and energy metabolism in aging and longevity.
